# Urban-Rural Disparity in Cardiac Implantable Electronic Device Use: A 10-Year Statewide Cohort

**DOI:** 10.5334/gh.1503

**Published:** 2025-12-10

**Authors:** Kenneth K. Cho, Edel O’Hagan, Brodie Sheahen, Sameer Karve, Natasha Nassar, Andrew Wilson, Tim Badgery-Parker, Clara K. Chow

**Affiliations:** 1 Westmead Applied Research Centre, Faculty of Medicine and Health, The University of Sydney, Entrance K, Level 5, Westmead Hospital, Hawkesbury Road, Westmead 2145, Australia; 2 Department of Cardiology, Orange Health Service, Orange 2800, New South Wales, Australia; 3 Faculty of Medicine and Health, School of Public Health, Menzies Centre for Health Policy, Charles Perkins Centre, The University of Sydney, Sydney, Australia

**Keywords:** Urban, Rural, Rurality, Cardiac devices, Cardiac implantable electronic devices

## Abstract

**Background:**

We examined cardiac implantable electronic device (CIED) implantation and outcomes related to geographical remoteness.

**Methods:**

This was a retrospective cohort study of adult cardiovascular hospitalisations in New South Wales, Australia (2008–2018). The primary outcome was CIED implantation in arrhythmia, cardiomyopathy, and syncope groups (and subcategories) among rural and regional residents. Secondary outcomes included CIED subtypes: pacemaker (PPM), implantable defibrillator (ICD) and cardiac resynchronisation therapy (CRT), examination of 10-year implant trends, and 5-year mortality rates adjusted for age and comorbidities.

**Results:**

Of the 1,291,258 cardiovascular admissions, 880,972 patients were from major cities (urban), 304,961 from inner regional (regional), and 105,325 from outer regional, rural, or remote areas (rural). Regional and rural patients received less PPMs (regional: aOR = 0.66; 95% CI 0.64–0.69; rural: aOR = 0.75; 95% CI 0.71–0.79), CRTs (regional: aOR = 0.71; 95% CI 0.65–0.78, rural: aOR = 0.72; 95% CI 0.83–0.94), and ICDs for regional patients (aOR = 0.72; 95% CI 0.67–0.77). Differences persisted in subcategories, including PPMs for complete heart block (regional: aOR = 0.58; 95% CI 0.56–0.60, rural: aOR = 0.66; 95% CI 0.62–0.70) and ICDs for ischaemic (regional: aOR = 0.44; 0.34–0.56, rural: aOR = 0.74; 95% CI 0.54–0.99) and non-ischaemic cardiomyopathy (regional: aOR = 0.64; 95% CI 0.56–0.73, rural: aOR = 0.72; 95% CI 0.59–0.87). Five-year mortality rates were higher in rural patients receiving PPM (urban = 23.7%; 95% CI23.2–24.2, rural = 26.4%; 95% CI 25.8–27.0), ICD (urban = 29.5%; 95% CI 28.2–30.7, rural = 32.5%; 95% CI 31.3–33.8) and CRT (urban = 24.2%; 95% CI 22.9–25.5, rural = 26.9%; 95% CI 25.5–28.3).

**Conclusion:**

Regional and rural patients had less CIED implantation, with higher 5-year rural mortality rates. Our study highlights the geographical disparity that occurs for patients requiring CIED and the need for further examination to determine the underlying causes and address these inequities.

## Introduction

Cardiac implantable electronic devices (CIEDs), including pacemakers (PPMs), implantable cardioverter defibrillators (ICDs), and cardiac resynchronisation therapy devices (CRTs), form the standard of care for treating patients with cardiomyopathy as well as symptomatic or life-threatening arrhythmia (1,2). Yet, translating gold-standard care to patients from regional and remote settings has proven difficult in cardiovascular disease globally. Patients from regional and rural areas experience a large and disproportionate burden of cardiovascular disease, with higher rates of cardiovascular risk factors, increased cardiovascular related hospitalisations, higher rates of cardiovascular mortality, and increased difficulty accessing cardiac procedures (e.g., with lower rates of coronary evaluation and revascularisation in patients with myocardial infarction) (3,4,5).

Data exploring differences in regional versus urban implant rates are limited to mainly small retrospective studies (6). Understanding whether such a disparity exists, and whether this is related to indication, comorbidities, or type of device, is important to ameliorate the urban-rural health divide. The aim of this study was to assess CIED implantation rates and outcomes by remoteness in a longitudinal cohort study of all hospitalised patients in New South Wales (NSW) Australia with arrhythmia, cardiomyopathy, or syncope, and to examine whether differences were related to age and comorbidities.

## Methods

### Study population

The study population comprised all people aged 18 years and over residing in NSW, Australia, with an acute public or private hospital admission for cardiovascular conditions from July 1, 2008 to June 30, 2018. The state of NSW has the largest population in Australia; in June 2021 this was estimated to be 8.2 million residents (32% of Australia).

### Data Sources

Data were sourced from linked NSW Health Admitted Patient Data Collection, Emergency Department Data Collection, and death records from the Registry of Births, Deaths, and Marriages. The linked dataset included a census of all inpatient admissions to public and private hospitals, public hospital emergency department presentations, and deaths registered in NSW. For each admission, sociodemographic data, clinical diagnoses and procedures performed, and patient status at discharge were recorded. Clinical diagnoses and procedures were classified using the International Classification of Diseases, Tenth Revision, Australian Modification (ICD-10-AM) and Australian Classification of Health Interventions, respectively. From the study population, we identified all people who underwent a cardiac implant, and using the principal diagnosis, we selected conditions where >10% of individuals had a cardiac implant (Table S1). Individuals were then classified into three diagnostic groups: (1) cardiac arrhythmia, (2) cardiomyopathy, and (3) syncope (Table S2). The cardiac arrhythmia group was subcategorised into complete heart block, other heart block, atrial fibrillation/atrial flutter, ventricular tachycardia/ventricular fibrillation/cardiac arrest (VT/VF/cardiac arrest), and sick sinus syndrome. The cardiomyopathy group was subcategorised into ischaemic and non-ischaemic cardiomyopathy.

Implantation was defined according to Australian Classification of Health Interventions procedure codes. Implantation of a left ventricular lead was used to identify CIEDs with CRT capability.

Remoteness was classified using the patients’ residential postcode with the Australian Statistical Geography Standard Remoteness Structure to categorize locations to major city (urban), inner regional (regional), or outer regional/rural/remote/very remote (rural) (7).

### Outcome

The primary outcome was implantation rate of a CIED, and secondary outcomes were implantation rates for pacemaker, ICD, or CRT in the arrhythmia, cardiomyopathy, and syncope diagnostic groups (and subcategories). We examined if implantation varied over the 10-year period and in emergency (based on care or treatment required within 24 hours, public hospitals only) versus nonemergency (elective) implantation. We also report by remoteness median days from admission to implant, median number of admissions from diagnosis to implant, median length of stay for implant insertion (days), and mortality rate per 100 patients at 30 days and 5 years.

### Data analysis

Descriptive analyses were conducted to assess frequency and rate of cardiac implants by cardiac condition and sociodemographic characteristics of each condition subtype. Baseline characteristics of patients from urban, regional and rural areas were compared using χ^2^ tests. We calculated adjusted odds ratios (aOR) for implantation of a device in regional and rural patients compared with urban patients, obtained from logistic regression models adjusting for age in years and indicators for each Elixhauser comorbidity (8), with comorbidities less relevant grouped as ‘other’. Additional covariates included sociodemographics and age category (18–44, 45–64, 65–74, 75–84, ≥85 years). Mortality rates were calculated using survival analysis and log-rank test applied to compare 30-day and 5-year mortality rates for patients who received a pacemaker, ICD, or CRT implant by each cardiac condition and stratified by remoteness. Time to implant was calculated using the median number of days of admissions before implant, and outcomes following implant including median length of stay in hospital were noted. All analyses were conducted in SAS V9.4 (Cary, NC) and R 4.3.3 (R Core Team, 2024). Authors TBP and CKC had full access to all data in the study and take responsibility for the integrity of the data and the accuracy of the data analysis.

### Ethics statement

The study was approved by the University of Sydney Human Research Ethics Committee. As it was a retrospective analysis of de-identified data, informed consent was not required.

## Results

The study population consisted of 1,291,258 patients with an acute cardiovascular condition (‘all patients’). Of these patients, 287,562 had a principal diagnosis of arrhythmia, cardiomyopathy, or syncope and 32,616 received a CIED. Of the total population, 68.2% of patients presented in an urban, 23.6% at a regional, and 8.2% at a rural centre. There were fewer females in rural areas compared with urban areas (urban: 46.06%; 95% CI 45.96–46.17, regional: 45.98%; 95% CI 45.81–46.16, rural: 43.89% 95% CI 43.60–44.19).

A greater proportion of patients from both regional and rural areas presented with arrythmia compared to urban (regional: 14.94%; 95% CI 14.82–15.07, rural: 14.90%; 95% CI 14.69–15.12, urban: 12.70%; 95% CI 12.63–12.77). This pattern was similar across diagnostic subcategories including complete heart block, other heart block, sick sinus syndrome, atrial fibrillation/flutter, ventricular tachycardia/fibrillation/cardiac arrest, and other arrhythmia. Similarly, a greater percentage of patients from both regional and rural areas presented with cardiomyopathy compared to urban areas (regional: 2.47%; 95% CI 2.42–2.53, rural: 2.51%; 95% CI 2.42–2.60, urban: 1.80%; 95% CI 1.77–1.83), and this was consistent across ischaemic and non-ischaemic subtypes. Syncope presentations also differed based on geography, but there was more syncope in urban areas (7.19%; 95% CI 7.13–7.24) compared with regional and rural sites (regional: 6.22; 95% CI 6.13–6.30, rural 5.76%; 95% CI 5.62–5.90) (Table 1).

**Table 1:** Characteristics of the cardiovascular hospitalisation population*.


	URBAN n = 880972% (95% CI)	REGIONAL n = 304961%; 95% CI	RURAL n = 105325%, 95% CI

Male	53.94 (53.83–54.04)	54.01 (53.84–54.19)	56.11 (55.81–56.40)

Female	46.06 (45.96–46.17)	45.98 (45.81–46.16)	43.89 (43.60–44.19)

Age, y			

18–44	12.55 (12.48–12.62)	9.22 (9.11–9.32)	9.46 (9.28–9.64)

45–64	28.69 (28.59–28.78)	27.22 (27.06–27.38)	29.32 (29.04–29.59)

65–74	20.15 (20.07–20.23)	23.06 (22.91–23.21)	24.06 (23.80–24.31)

75–84	22.92 (22.83–23.00)	25.36 (25.20–25.51)	24.41 (24.15–24.67)

85+	15.70 (15.62–15.78)	15.15 (15.02–15.27)	12.76 (12.56–12.96)

Comorbidities			

Cancer	0.01 (0.01–0.02)	0.01 (0.00–0.01)	0.01 (0.01–0.02)

Congestive heart failure	0.29 (0.28–0.30)	0.17 (0.16–0.19)	0.19 (0.16–0.21)

Diabetes	0.51 (0.49–0.52)	0.30 (0.28–0.32)	0.36 (0.32–0.40)

Hypertension	0.55 (0.53–0.56)	0.34 (0.32–0.37)	0.38 (0.35–0.42)

Obesity	0.02 (0.01–0.02)	0.02 (0.02–0.03)	0.02 (0.01–0.03)

Renal failure	0.19 (0.18–0.20)	0.10 (0.09–0.11)	0.11 (0.09–0.13)

Other comorbidity	0.55 (0.53–0.56)	0.29 (0.27–0.31)	0.28 (0.25–0.31)

Diagnostic group			

Arrythmia diagnosis	12.70 (12.63–12.77)	14.94 (14.82–15.07)	14.90 (14.69–15.12)

Complete heart block	0.51 (0.50–0.53)	0.60 (0.58–0.63)	0.64 (0.59–0.69)

Other heart block	0.55 (0.54–0.57)	0.68 (0.65–0.71)	0.70 (0.65–0.75)

Sick sinus syndrome	0.50 (0.49–0.52)	0.53 (0.51–0.56)	0.55 (0.51–0.60)

Atrial fibrillation/flutter	8.78 (8.72–8.83)	10.76 (10.65–10.87)	10.50 (10.32–10.69)

Ventricular tachycardia/ventricular fibrillation/cardiac arrest	1.22 (1.20–1.24)	1.14 (1.10–1.18)	1.16 (1.10–1.22)

Other arrythmia	1.23 (1.21–1.25)	1.48 (1.44–1.52)	1.69 (1.62–1.77)

Cardiomyopathy diagnosis	1.80 (1.77–1.83)	2.47 (2.42–2.53)	2.51 (2.42–2.60)

Ischaemic	0.38 (0.36–0.39)	0.49 (0.46–0.51)	0.47 (0.43–0.51)

Non-ischaemic	1.70 (1.67–1.73)	2.35 (2.30–2.41)	2.33 (2.24–2.43)

Syncope Diagnosis	7.19 (7.13–7.24)	6.22 (6.13–6.30)	5.76 (5.62–5.90)


*All patients aged over 18 years who presented with selected cardiovascular conditions to a hospital in New South Wales from 2008 to 2018 (see Table S1 for International Classification of Diseases, Tenth Revision, Australian Modification codes). Percentages provided are of total patients from urban, regional and rural areas, respectively.

There were 32,616 total CIEDs implanted; most CIEDs were implanted in urban areas (n = 24,303), where a higher proportion of patients received devices (2.76%; 95% CI, 2.71–2.82) compared with regional (n = 5975, 1.96%; 95% CI 1.88–2.04) and rural areas (n = 2338, 2.23%; 95% CI 2.08–2.38). PPMs were the most common device implanted (n = 22,543), and urban patients had higher implant rates (n = 16,897; 1.92%; 95% CI 1.89–1.95) than patients in regional (n = 4124, 1.35%; 95% CI 1.31–1.39) and rural settings (n = 1522, 1.45%; 95% CI 1.37–1.52). ICDs (n = 6866, 23.4%; 95% CI, 22.9–23.9 of all CIEDs) were also implanted at higher rates in urban patients (n = 5044, 0.57%; 95% CI 0.56–0.59) versus patients from regional centres (n = 1244, 0.41%; 95% CI 0.39–0.43) and rural areas (n = 578, 0.55%; 95% CI 0.51–0.60). Similarly, CRTs (n = 3207, 11%; 95% CI, 10.7–11.4 of CIEDs) were implanted at higher rates in urban (n = 2362, 0.27%; 95% CI 0.26–0.28) versus regional (n = 607, 0.20%; 95% CI 0.18–0.22) and rural areas (n = 238, 0.23%; 95% CI 0.20–0.26) (Table 2).

**Table 2:** Cardiac device implants in the cardiovascular hospitalisation population*.


	URBAN n (%, 95% CI)	REGIONAL n (%, 95% CI)	RURAL n (%, 95% CI)

Cardiac device implant	24303 (2.76, 2.71–2.82)	5975 (1.96, 1.88–2.04)	2338 (2.23, 2.08–2.38)

Pacemakers	16897 (1.92, 1.89–1.95)	4124 (1.35, 1.31–1.39)	1522 (1.45, 1.37–1.52)

Implantable cardiac defibrillators	5044 (0.57, 0.56–0.59)	1244 (0.41, 0.39–0.43)	578 (0.55, 0.51–0.60)

Cardiac resynchronisation therapy	2362 (0.27, 0.26–0.28)	607 (0.20, 0.18–0.22)	238 (0.23, 0.20–0.26)


*All patients aged over 18 years who presented with selected cardiovascular conditions to a hospital in New South Wales from 2008 to 2018 (see Table S1 for International Classification of Diseases, Tenth Revision, Australian Modification codes). Percentages provided are of total patients from urban, regional and rural areas, respectively.

### Device implants by type and primary diagnoses, analysed through remoteness

**Figure 1:**
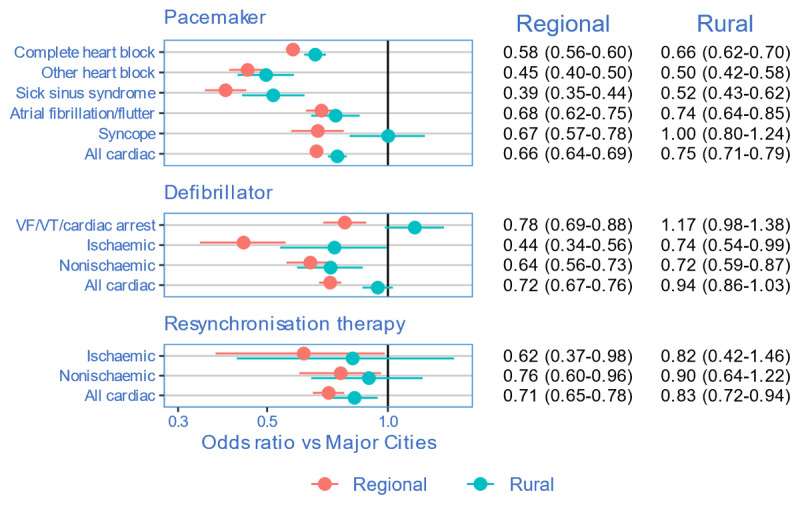
Adjusted odds ratio with 95% CI for CIEDs in regional and rural compared to urban (reference) locations. Adjusted for age and comorbidities. Odds ratio <1 suggests lower implantation rates in regional and rural compared to urban locations.

For each device and cardiac diagnosis subcategory, patients from regional and rural areas consistently had lower implant rates than their urban counterparts, which persisted after adjusting for age and comorbidities (Figure 1). Patients from regional or rural areas were less likely to have PPM (regional: aOR 0.66; 95% CI 0.64–0.69, rural: aOR 0.75; 95% CI 0.71–0.79) and CRT (regional: aOR 0.71; 95% CI 0.65–0.78, rural: aOR 0.72; 95% CI 0.83–0.94), whereas patients were less likely to have ICD in regional (aOR 0.72; 95% CI 0.67–0.77) but not rural areas (aOR 0.94; 95% CI 0.86–1.03).

With urban patients as reference, regional and rural patients were less likely to receive PPM for complete heart block (regional: aOR 0.58; 95% CI 0.56–0.60, rural aOR: 0.66; 95% CI 0.62–0.70), other heart block, (regional: aOR 0.45; 95% CI 0.4–0.50, rural: aOR 0.5; 95% CI 0.42, 0.58), sick sinus syndrome, (regional: aOR 0.39; 95% CI 0.35–0.44, rural: aOR 0.52; 95% CI 0.43–0.62), and atrial fibrillation/flutter (regional: aOR 0.68; 95% CI 0.63–0.75, rural: aOR 0.74; 95% CI 0.64–0.85), wheras patients were less likely to receive PPM for syncope inregional centres (aOR 0.67; 95% CI 0.57–0.78) but not rural (aOR 1; 95% CI 0.8–1.24).

Rural and regional patients were also less likely to receive ICDs for cardiomyopathies including both ischaemic (regional: aOR 0.44; 95% CI0.34–0.56, rural: aOR 0.74; 95% CI0.54–0.99) and non-ischaemic subtypes (regional: aOR 0.64; 95% CI 0.56–0.73, rural: aOR 0.72; 95% CI 0.59–0.87). Patients with VF/VT/cardiac arrest from regional areas were less likely to receive an ICD (aOR 0.78; 95% CI 0.69–0.88), but not those from rural locations (aOR 1.17; 95% CI 0.98–1.38), again noting the low numbers for these estimates.

There were fewer CRTs for patients in regional areas with ischaemic cardiomyopathy (regional: aOR 0.62; 95% CI 0.37–0.98) and non-ischaemic cardiomyopathy (regional: aOR 0.76; 95% CI 0.6–0.96), noting the wide confidence intervals for patients with these subsets from rural areas (ischaemic: aOR 0.82; 95% CI 0.42–1.46), non-ischaemic: aOR 0.9; 95% CI 0.64–1.22).

**Figure 2:**
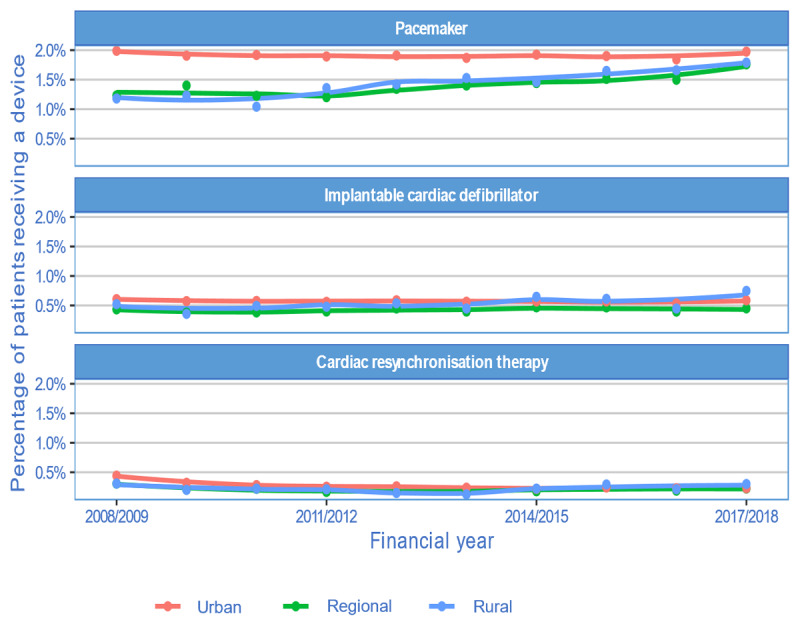
Trends in CIED insertion by remoteness.

On review of 10-year trends, the proportions of patients receiving pacemakers from regional and rural areas appear to be increasing, without notable changes for ICDs and CRTs (Figure 2).

### Acuity and clinical outcomes of implantation of cardiac devices

People residing in rural areas were less likely to be emergent cases compared with people from regional and major cities. Emergency status differed for people presenting with an arrhythmia and having a PPM, from 67% of people from urban, 48% from regional, and 36% from regional areas. Emergency status for cardiomyopathy was lower across regional and rural regions for patients subsequently receiving an ICD with emergent rates of 23%, 21% and 20%, respectively. Length of stay in hospital was either similar or shorter for those from outer regions compared with major cities (Table S3).

### Mortality at 30 days and 5 years in patients from urban versus regional and rural areas

Mortality rates among patients receiving devices were higher in rural and regional areas compared to urban areas (Table 3). Patients from regional and rural areas had higher 5-year rates of mortality compared with urban patients, including regional patients receiving PPMs (urban: 23.7%; 95% CI 23.2–24.2, regional: 25.0%; 95% CI 24.5–25.6), and rural receiving PPM (26.4%; 95% CI 25.8–27.0), ICD (urban: 29.5%; 95% CI 28.2–30.7, rural: 32.5%; 95% CI 31.3–33.8) and CRT (urban: 24.2%; 95% CI 22.9–25.5, rural: 26.9%; 95% CI 25.5–28.3).

**Table 3:** Adjusted* mortality rates (95% CI) at 30 days and five years after cardiac implantation for patients at urban versus regional and rural locations.


REMOTENESS CATEGORY	PACEMAKER %, (95% CI), P VALUE**	DEFIBRILLATOR %, (95% CI), P VALUE**	CARDIAC RESYNCHRONISATION %, (95% CI), P VALUE**

30-day mortality			

Urban	0.6 (0.5–0.7)	0.9 (0.6–1.2)	0.8 (0.5–1.1)

Regional	0.7 (0.6–0.8), P = 0.5	1.0 (0.7–1.3), P = 0.8	0.8 (0.5–1.1), P = 0.8

Rural	0.7 (0.6–0.8), P = 0.15	1.1 (0.7–1.4), P = 0.5	0.9 (0.6–1.2), P = 0.5

5-year mortality			

Urban	23.7 (23.2–24.2)	29.5 (28.2–30.7)	24.2 (22.9–25.5)

Regional	25.0 (24.5–25.6), P = 0.001	30.9 (29.6–32.2), P = 0.12	25.5 (24.2–26.8), P = 0.17

Rural	26.4 (25.8–27.0), P < 0.001	32.5 (31.1–33.8), P = 0.002	26.9 (25.5–28.3), P = 0.005


*Rates are adjusted for age and presence of Elixhauser comorbidities. **P value for mortality in remoteness category vs urban location.

## Discussion

This is the largest study to systematically examine implant rates for urban versus regional and rural areas, stratified by implant diagnosis and adjusted for age and comorbidities.

### Urban-rural disparity in CIED implantation rates

**Figure 3:**
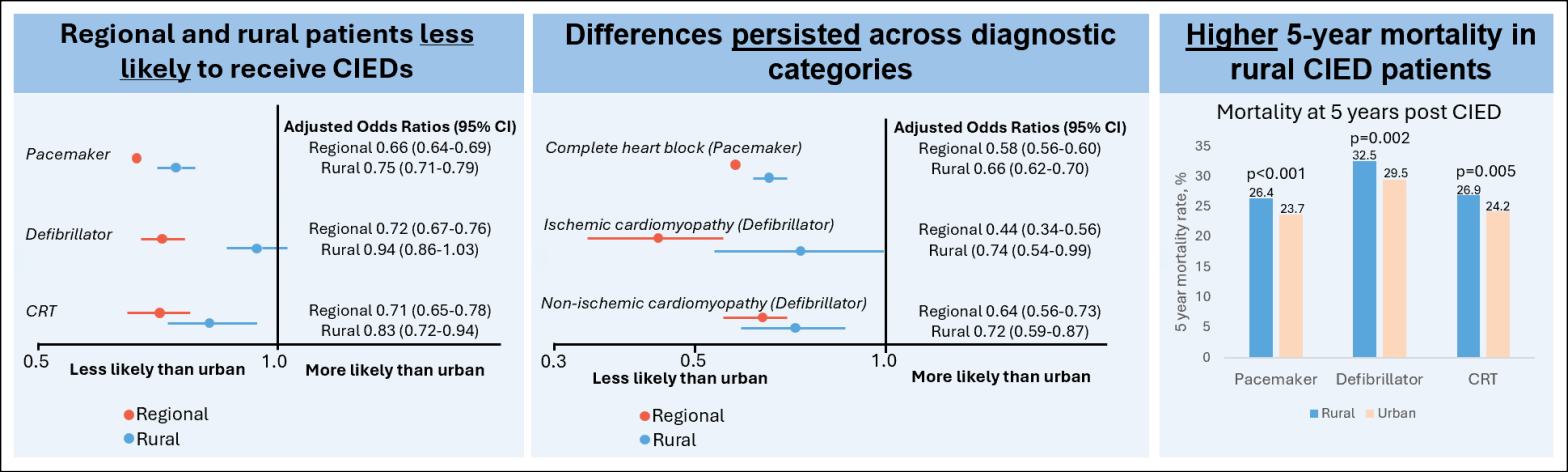
Urban-rural disparity in cardiac implantable electronic device use: 10-year state-wide cohort of 32,616 consecutive CIED patients.

Our major finding was patients in urban areas had higher rates of CIED implantation and were more likely to receive CIEDs, when adjusted for age and comorbidities, compared with patients from regional and rural locations. This finding persisted in important diagnostic subsets, including decreased likelihood of PPMs for complete heart block as well as ICDs and CRTs for cardiomyopathy, especially relevant given these indications are prognostic and highlighted in international guidelines (2,9) (Figure 3).

Few studies have explored the urban-rural disparity for CIEDs. A retrospective observational study of three centres (two rural and one urban) in Canada found decreased ICD implantation rates for patients from regional/rural centres, and suggested reasons may include lower rates of referrals and patient refusal. Of their 335 ICD-eligible patients (240 urban, 95 rural), referral rates were higher from the urban centre compared with the rural institutions (80.4% vs 68.3%; P = 0.024, urban location OR 1.81; 95% CI 1.01–3.26) and patient refusal rates for referral were higher in the rural locations (13.7% vs 2.1%; P < 0.0001) (10). A separate explanation may involve the proximity to an implantation centre, with a study in Germany suggesting areas served by an electrophysiology centre have higher implant rates than areas served by general hospitals, with regional centres less likely to implant ICDs for primary prevention of sudden cardiac death (11). A lack of local resources may be a contributing factor in the Australian context, and this is supported by consensus documents which suggest CIED implantation centres in regional and rural areas are highly variable and at times deficient, with a recommendation to establish local CIED implantation and CIED clinic services (12).

### Urban-rural disparity in 5-year survival after CIED implantation

Our second major finding was patients from regional and rural areas had higher mortality rates compared with their urban counterparts, and in CIED patients there were increased 5-year mortality rates for non-urban patients receiving PPMs and for rural patients receiving ICDs and CRTs.

Non-urban patients with cardiovascular disease often experience poorer health outcomes, with cited contributing factors including differences in risk factor and comorbid burden, financial constraints, and use of or access to health services, with studies mainly focused on coronary artery disease or heart failure cohorts (13,14). However, studies suggest these poorer outcomes are not completely attenuated despite mediating for age, sex, type of myocardial infarction (STEMI, NSTEMI, MI unspecified), socioeconomic status, insurance status, emergency presentation, individual comorbidities, and revascularisation (14,15,16). Although variations in models exist, consensus documents suggest PPMs are now are often managed by rural cardiologists, whereas CRT devices are commonly managed by visiting specialist cardiac electrophysiologists (12). Of interest, a more contemporary dataset may help review the impact of increased pacemaker implants in regional and rural areas that were noted in the 10-year trends and whether this improves the urban-rural disparity for these patients’ outcomes. When reviewing the broader literature, there was a scarcity of research exploring survival outcomes of urban versus rural CIED patients.

### Study limitations

This study has several limitations. First, our study used a hospital coded database, and as such confounding and uncoded variables may have existed that were evident to the treating clinician but were unable to be identified in our analysis. For example, although controlling for comorbidities, our study was unable to assess a patient’s functional capacity, which may have affected clinician decision-making. Similarly, our study cannot examine the appropriateness of CIED implantation, including the possibility of overtreatment in the urban compared to the regional and rural cohorts. Additionally, although we were able to demonstrate disparity across geographical zones that persisted despite adjustment for age and comorbidities, we were unable to evaluate further covariates or risk factors underlying the observed disparities in CIED implantation rates and mortality. Finally, with regard to implant numbers, we did not differentiate between generator changes for end-of-life battery and a new device implant, and hence the actual number of device implantations performed in NSW may have been lower than described. Nevertheless, these data allow a reasonable estimate of the relative percentage and implant rates of the device types, with studies suggesting that ICD-10 administrative coding is reasonably robust when compared with registries (17).

## Conclusion

This analysis of a large-scale and real-world dataset demonstrates the existence of decreased implant rates of CIEDs in regional and rural patients and generally poorer mortality outcomes. As the cardiac devices provide select patients with symptomatic and survival benefit, this research should provoke further examination for underlying contributing factors influencing the CIED urban-rural divide. This research should also provoke greater examination and monitoring of urban-rural differences, with research to address these inequities.

## Additional File

The additional file for this article can be found as follows:

10.5334/gh.1503.s1 Supplementary File. Tables S1–S3.

## Abbreviations

Non-standard abbreviations and acronyms: cardiac implantable electronic device (CIED), pacemaker (PPM), implantable cardioverter defibrillator (ICD), cardiac resynchronisation therapy device (CRT), New South Wales (NSW), International Classification of Diseases, Tenth Revision, Australian Modification (ICD-10-AM), adjusted odds ratio (aOR)
