# Supplementary material for: Urban-Rural Disparity in Cardiac Implantable Electronic Device Use: A 10-Year Statewide Cohort
Source: Glob Heart. 2025 Dec 10;20(1):109. doi: 10.5334/gh.1503 (PMC12700143; doi:10.5334/gh.1503)
Supplement: Supplementary File. — Tables S1–S3. [file gh-20-1-1503-s1.pdf]

30-day mortality			
Urban	0.6 (0.5-0.7)	0.9 (0.6-1.2)	0.8 (0.5-1.1)
Regional	0.7 (0.6-0.8), P=0.5	1.0 (0.7-1.3), P=0.8	0.8 (0.5-1.1), P=0.8
Rural	0.7 (0.6-0.8), P=0.15	1.1 (0.7-1.4), P=0.5	0.9 (0.6-1.2), P=0.5
5-year mortality			
Urban	23.7 (23.2-24.2)	29.5 (28.2-30.7)	24.2 (22.9-25.5)
Regional	25.0 (24.5-25.6), P=0.001	30.9 (29.6-32.2), P=0.12	25.5 (24.2-26.8), P=0.17
Rural	26.4 (25.8-27.0), P<0.001	32.5 (31.1-33.8), P=0.002	26.9 (25.5-28.3), P=0.005

*Rates are adjusted for age and presence of Elixhauser comorbidities.
**P value for mortality in remoteness category vs urban location

Supplement

Table S1. International Classification of Diseases 10th revision Australian Modification (ICD10AM) codes for all patients included in the study

ICD10AM code and description
B33.2 Viral carditis
E11.53 Type 2 diabetes mellitus with diabetic cardiomyopathy
I07.1 Tricuspid insufficiency
I08.1 Disorders of both mitral and tricuspid valves
I08.3 Combined disorders of mitral, aortic and tricuspid valves
I10 Essential (primary) hypertension
I20.0 Unstable angina
I20.1 Angina pectoris with documented spasm
I20.8 Other forms of angina pectoris
I20.9 Angina pectoris, unspecified
I21.0 Acute transmural myocardial infarction of anterior wall
I21.1 Acute transmural myocardial infarction of inferior wall
I21.2 Acute transmural myocardial infarction of other sites
I21.3 Acute transmural myocardial infarction of unspecified site
I21.4 Acute subendocardial myocardial infarction
I21.9 Acute myocardial infarction, unspecified
I23.8 Other current complications following acute myocardial infarction
I25.10 Atherosclerotic heart disease, of unspecified vessel
I25.11 Atherosclerotic heart disease, of native coronary artery
I25.5 Ischaemic cardiomyopathy
I25.8 Other forms of chronic ischaemic heart disease
I25.9 Chronic ischaemic heart disease, unspecified
I30.9 Acute pericarditis, unspecified
I33.0 Acute and subacute infective endocarditis
I34.0 Mitral (valve) insufficiency
I35.0 Aortic (valve) stenosis
I35.1 Aortic (valve) insufficiency
I35.9 Aortic valve disorder, unspecified
I42.0 Dilated cardiomyopathy
I42.1 Obstructive hypertrophic cardiomyopathy
I42.2 Other hypertrophic cardiomyopathy
I42.4 Endocardial fibroelastosis
I42.6 Alcoholic cardiomyopathy
I42.7 Cardiomyopathy due to drugs and other external agents
I42.8 Other cardiomyopathies
I42.9 Cardiomyopathy, unspecified
I43.0 Cardiomyopathy in infectious and parasitic diseases classified elsewhere
I44.0 Atrioventricular block, first degree
I44.1 Atrioventricular block, second degree
I44.2 Atrioventricular block, complete
I44.3 Other and unspecified atrioventricular block
I44.4 Left anterior fascicular block
I44.7 Left bundle-branch block, unspecified
I45.1 Other and unspecified right bundle-branch block
I45.2 Bifascicular block

I45.3 Trifascicular block
I45.4 Nonspecific intraventricular block
I45.5 Other specified heart block
I45.6 Pre-excitation syndrome
I45.8 Other specified conduction disorders
I45.9 Conduction disorder, unspecified
I46.0 Cardiac arrest with successful resuscitation
I46.9 Cardiac arrest, unspecified
I47.1 Supraventricular tachycardia
I47.2 Ventricular tachycardia
I47.9 Paroxysmal tachycardia, unspecified
I48 Atrial fibrillation and flutter
I48.0 Paroxysmal atrial fibrillation
I48.1 Persistent atrial fibrillation
I48.2 Chronic atrial fibrillation
I48.3 Typical atrial flutter
I48.4 Atypical atrial flutter
I48.9 Atrial fibrillation and atrial flutter, unspecified
I49.0 Ventricular fibrillation and flutter
I49.2 Junctional premature depolarization
I49.3 Ventricular premature depolarization
I49.4 Other and unspecified premature depolarization
I49.5 Sick sinus syndrome
I49.8 Other specified cardiac arrhythmias
I49.9 Cardiac arrhythmia, unspecified
I50.0 Congestive heart failure
I50.1 Left ventricular failure (if secondary diagnosis is coronary heart disease)
I50.1 Left ventricular failure (with secondary diagnosis of no coronary heart disease)
I50.9 Heart failure, unspecified
I51.6 Cardiovascular disease, unspecified
I51.7 Cardiomegaly
I51.8 Other ill-defined heart diseases
I51.9 Heart disease, unspecified
I63.3 Cerebral infarction due to thrombosis of cerebral arteries
I63.4 Cerebral infarction due to embolism of cerebral arteries
I63.9 Cerebral infarction, unspecified
I64 Stroke, not specified as hemorrhage or infarction
I95.1 Orthostatic hypotension
I95.9 Hypotension, unspecified
I97.1 Other functional disturbances following cardiac surgery
I97.8 Other intraoperative and postprocedural disorders of circulatory system, not elsewhere classified
O99.4 Diseases of the circulatory system in pregnancy, childbirth and the puerperium
Q24.6 Congenital heart block
R00.0 Tachycardia, unspecified
R00.1 Bradycardia, unspecified
R00.2 Palpitations
R00.8 Other and unspecified abnormalities of heart beat
R06.0 Dyspnea
R07.3 Other chest pain
R07.4 Chest pain, unspecified
R29.6 Tendency to fall, not elsewhere classified
R41.0 Disorientation, unspecified
R42 Dizziness and giddiness
R55 Syncope and collapse
R56.8 Other and unspecified convulsions
R57.0 Cardiogenic shock
R94.3 Abnormal results of cardiovascular function studies
S06.02 Loss of consciousness of brief duration [less than 30 minutes]
S06.5 Traumatic subdural hemorrhage

S06.6 Traumatic subarachnoid hemorrhage
T82.1 Mechanical complication of cardiac electronic device
T82.5 Mechanical complication of other cardiac and vascular devices and implants
T82.6 Infection and inflammatory reaction due to cardiac valve prosthesis
T82.7 Infection and inflammatory reaction due to cardiac and vascular devices, implants and grafts, not elsewhere classified
T82.8 Other specified complications of cardiac and vascular devices, implants and grafts
T86.2 Heart transplant failure and rejection
Z45.0 Adjustment and management of cardiac device
Abbreviations: ICD10AM International Classification of Diseases 10th revision Australian Modification.

Table S2. International Classification of Diseases 10th revision Australian Modification (ICD10AM) codes for the arrhythmia, cardiomyopathy, and syncope diagnostic groups

Diagnostic group	Sub-catergory	ICD10AM code	ICD10AM description
Arrythmia	Complete heart block	I44.2	Atrioventricular block, complete
	Other heart block	I44.0	Atrioventricular block, first degree
		I44.1	Atrioventricular block, second degree
		I44.3	Other and unspecified atrioventricular block
		I44.4	Left anterior fascicular block
		I44.7	Left bundle-branch block, unspecified
		I45.1	Other and unspecified right bundle-branch block
		I45.2	Bifascicular block
		I45.3	Trifascicular block
		I45.4	Nonspecific intraventricular block
		I45.5	Other specified heart block
		I45.8	Other specified conduction disorders
		I45.9	Conduction disorder, unspecified
	Sick sinus syndrome	I49.5	Sick sinus syndrome
	Atrial fibrillation / atrial flutter	I48	Atrial fibrillation and flutter
		I48.0	Paroxysmal atrial fibrillation
		I48.1	Persistent atrial fibrillation
		I48.2	Chronic atrial fibrillation
		I48.9	Atrial fibrillation and atrial flutter, unspecified
	VT/VF/Cardiac arrest	I46.0	Cardiac arrest with successful resuscitation
		I47.2	Ventricular tachycardia
		I49.0	Ventricular fibrillation and flutter
	Other arrythmia	I49.2	Junctional premature depolarization

		I49.8	Other specified cardiac arrhythmias
		I49.9	Cardiac arrhythmia, unspecified
		Q24.6	Congenital heart block
		R00.1	Bradycardia, unspecified
Cardiomyopathy	Ischaemic	I25.5	Ischaemic cardiomyopathy
		I50.1	Left ventricular failure (if any secondary diagnosis is coronary heart disease)
	Non-ischaemic	E11.53	Type 2 diabetes mellitus with diabetic cardiomyopathy
		I42.0	Dilated cardiomyopathy
		I42.1	Obstructive hypertrophic cardiomyopathy
		I42.2	Other hypertrophic cardiomyopathy
		I42.6	Alcoholic cardiomyopathy
		I42.7	Cardiomyopathy due to drugs and other external
		I42.8	Other cardiomyopathies
		I42.9	Cardiomyopathy, unspecified
		I43.0	Cardiomyopathy in infectious and parasitic diseases
		I50.1	Left ventricular failure (with no secondary diagnosis of coronary heart disease)
Syncope		R55	Syncope and collapse

Abbreviations: ICD10AM International Classification of Diseases 10th revision Australian Modification, VF ventricular fibrillation, VT ventricular tachycardia.

Table S3: Procedural status of cardiac device implantation for patients at an urban versus regional and rural areas

	PPM			ICD		
	Urban	Regional	Rural	Urban	Regional	Rural
**All cardiac** Emergent	11201/16897 (65.3%)	2394/4124 (45.3%)	85/15225 (34.4%)	2586/5044 (50.3%)	563/1244 (35.4%)	256/578 (27.9%)
Median days to implant	1	2	2	0	0	0
Median admissions to device	1	1	2	2	2	2
Median length of stay (days)	4	2	1	4	1	1
Mean length of stay (days)	6.1 (8.9)	2.9 (5.1)	2 (4)	7.5 (10.3)	3.4 (5.8)	2.3 (5.2)
**Arrhythmia** Emergent	9345/13783 (66.8%)	2120/3462 (48.1%)	759/1280 (36.1%)	1498/2217 (66.3%)	360/591 (48.1%)	165/282 (37.1%)
Median days to implant	1	2	2	2	3	3.5
Median admissions to device	1	1	1	1	1	1

Median length of stay (days)	4	2	1	7	2	1
Mean length of stay (days)	5.4 (7.8)	2.5 (4.2)	1.7 (3)	8.5 (9.9)	3.8 (5.7)	2.2 (4.3)
**Complete heart block** Emergent	2755/3237 (84.0%)	865/1074 (68.0%)	328/411 (50.2%)	93/105 (86.5%)	23/25 (72.1%)	9 /10 (50.1%)
Median days to implant	1	3	3	2	2.5	4
Median admissions to device	1	1	1	1	1	1
Median length of stay (days)	4	2	1	6	1.5	1
Mean length of stay (days)	5.8 (11)	2.6 (4.7)	1.5 (2.7)	7.5 (8.3)	2.3 (3.9)	2.2 (3.1)
**Other heart block** Emergent	1845/2780 (65.4%)	478/785 (48.6%)	173/296 (35.9%)	70/156 (50.3%)	22/39 (45.6%)	6/21 (18.9%)
Median days to implant	1	2	2	0	0	0
Median admissions to device	1	1	1	1	1	1
Median length of stay (days)	3	2	1	2	1	1
Mean length of stay (days)	4.6 (5.6)	2.4 (3.5)	1.6 (2.8)	5.2 (6.8)	2.5 (3.6)	0.8 (1.6)
**Sick sinus syndrome** Emergent	2097/3201 (64.6%)	530/826 (49.8%)	212/337 (38.3%)	48/69 (69.5%)	15/17 (76.9%)	7/8 (52.8%)
Median days to implant	1	3	4	4	3	3.5
Median admissions to device	1	1	1	1	1	1
Median length of stay (days)	4	2	2	5	3	1
Mean length of stay (days)	5.5 (6.1)	3 (4.2)	2 (3.2)	7 (8)	3.2 (3.7)	1.6 (3.3)
**Atrial fibrillation/flutter** Emergent	1393/2394 (57.1%)	317/685 (37.0%)	114/236 (29.7%)	138/294 (46.0%)	43/103 (33.6%)	13/43 (17.4%)
Median days to implant	0	0	1	0	0	0
Median admissions to device	1	1	1	1	1	1
Median length of stay (days)	4	1	1	3	1	1
Mean length of stay (days)	5.6 (7.4)	2.7 (4.4)	1.8 (3.1)	6.2 (8.2)	3.2 (5.6)	1.3 (2.4)
**VF/VT/cardiac arrest** Emergent	186/234 (78.1%)	50/57 (75.1%)	17/22 (46.6%)	1102/1465 (73.7%)	265/376 (55.7%)	135/186 (48.3%)
Median days to implant	2	5	5.5	4	6	6
Median admissions to device	1	1	1	1	1	1
Median length of stay (days)	7	4	1	8	3	2
Mean length of stay (days)	9.5 (10.1)	4 (4.3)	2.1 (7.2)	9.7 (10.4)	4.5 (6.1)	2.8 (5)
**Other arrhythmia**	1628/2505 (63.8%)	348/579 (45.8%)	114/228 (37.9%)	103/210 (49.0%)	24/66 (28.5%)	9/28 (18.3%)

Emergent						
Median days to implant	1	2	3	0	0	0
Median admissions to device	1	1	1	1	1	1
Median length of stay (days)	3	1	1	3	1	1
Mean length of stay (days)	5 (6.4)	2.2 (3.4)	1.6 (3)	6.1 (8.3)	3.1 (6.6)	1.2 (2.3)
**Cardiomyopathy** Emergent	118/294 (39.3%)	29/94 (24.0%)	21/41 (18.4%)	335/1436 (22.9%)	109/407 (20.8%)	64/205 (19.9%)
Median days to implant	0	0	0	0	0	0
Median admissions to device	1	1	1	1	1	1
Median length of stay (days)	2	1	1	1	1	1
Mean length of stay (days)	6.8 (10.6)	3 (7.2)	1.9 (4.5)	4 (7)	2.2 (4.3)	2 (4.8)
**Ischaemic cardiomyopathy** Emergent	34/73 (46.2%)	4/17 (20.1%)	4/11 (25.3%)	89/453 (19.5%)	28/98 (22.4%)	14/65 (14.1%)
Median days to implant	0	0	0	0	0	0
Median admissions to device	1	1	1	1	1	1
Median length of stay (days)	2	1	1	1	1	1
Mean length of stay (days)	6.5 (8)	2.5 (3.9)	1 (2.2)	3.6 (6.7)	2.4 (4.2)	1.9 (5.9)
**Non-ischaemic** Emergent	101/239 (41.2%)	27/79 (26.3%)	10/33 (19.0%)	257/1009 (24.9%)	88/319 (21.4%)	5/1433 (23.2%)
Median days to implant	0	0	0	0	0	0
Median admissions to device	1	1	1	1	1	1
Median length of stay (days)	2	1	1	1	1	1
Mean length of stay (days)	7.5 (11.5)	3.1 (7.6)	2.2 (5)	4.2 (7.2)	2.4 (4.4)	1.9 (4.1)
**Syncope** Emergent	732/1003 (71.6%)	140/211 (49.7%)	70/94 (46.4%)	114/138 (81.4%)	23/30 (62.0%)	12/15 (46.4%)
Median days to implant	1	2	3	5	8	7
Median admissions to device	1	1	1	1	1	1
Median length of stay (days)	3	2	2	8	6	3
Mean length of stay (days)	5 (5.6)	2.7 (3.9)	1.8 (2.6)	7.9 (6.4)	5.8 (7.2)	2.6 (4.2)
